# Autologous fat grafting in the treatment of a scleroderma stump-skin ulcer: a case report

**DOI:** 10.1080/23320885.2021.1881521

**Published:** 2021-02-12

**Authors:** Dilia Giuggioli, Amelia Spinella, Emanuele Cocchiara, Marco de Pinto, Massimo Pinelli, Luca Parenti, Carlo Salvarani, Giorgio De Santis

**Affiliations:** a Scleroderma Unit, University of Modena and Reggio Emilia, Medical School, Azienda Ospedaliero-Universitaria, Policlinico di Modena, Modena, Italy; b Rheumatology Unit, University of Modena and Reggio Emilia, Medical School, Azienda Ospedaliero-Universitaria, Policlinico di Modena, Modena, Italy; c Rheumatology Unit, ASMN Reggio Emilia, Reggio Emilia, Italy; d Plastic Surgery Unit, Azienda Ospedaliero-Universitaria, Policlinico di Modena, Modena, Italy; e Department of Medical and Surgical Sciences, University of Modena and Reggio Emilia, Azienda Ospedaliero-Universitaria, Policlinico di Modena, Modena, Italy

**Keywords:** systemic sclerosis, stump-digital ulcers, autologous fat grafting

## Abstract

Here we describe the case of a 60‐year‐old-woman with systemic sclerosis sent to our Scleroderma Unit to treat digital stumps. The stumps were successfully treated with autologous fat grafting (crown-shape infiltration). Our technique of autologous lipotransfer improved wound healing in a scleroderma patient with stump-digital ulcers where all other options failed.

## Introduction

Digital ulcers (DUs) represent one of the most severe and frequent manifestations of systemic sclerosis (SSc) occurring in almost 50% of SSc patients [1–7]. Moreover, DUs are painful, recurrent, hard to heal, they can become infected and evolve into gangrene, risking amputation [8]. This surgical technique results in a negative impact on digital function and deeply affect patients’ quality of life. Taking into account that skin ulcers and amputations represent a clinical challenge, a combined treatment (medical, surgical and physiotherapy) is mandatory. The adipose tissue is recognised as an easy to access source, and stem cell-based therapies have emerged as a promising option to improve DUs healing too because of their broad range of clinical applications [9–14]. Autologous fat grafting (AFG) provides trophic effects and could have beneficial effects for the treatment of SSc skin ulcers. Up to now, there is no data on the treatment of amputation in scleroderma patients.

We are reporting a case of a SSc patient treated with AFG injection for the treatment of long term stump skin lesions arriving at the 24-week follow up period.

## Case presentation

A 60‐year‐old woman affected by SSc with recurrent digital ulcers was sent to our Scleroderma Unit for the treatment of the amputation stumps resistant to traditional therapies. The disease was characterised by Raynaud phenomenon, telengiectasias on hands and face, oesophageal and interstitial lung involvement. She was positive to serum antinucleolar antibodies and presented a capillaroscopic scleroderma pattern. During 2018, the patient developed multiple DUs unresponsive to systemic therapies (calcium-channel blockers, prostanoids and/or anti-ET receptors). The ulcer on the II finger of the right hand became particularly severe, showing irregular margins with exposure of both the flexor tendon and phalangeal bone, and even the digital ulcer on the III finger worsened. Amputation of both distal and proximal phalanges of the II finger together with the amputation of the distal phalanges of the III finger was performed in a surgical department. Despite topical therapy and antibiotics (i.e. co-amoxiclav, flucloxacillin and ciprofloxacin) used to treat the recurrent infections, the DUs on the stumps continued to increase in size and depth with exudate, and became more painful (VAS: 9/10) even though analgesics (paracetamol and opioids) were used. 3 months following surgical intervention, a further amputation was proposed, and as a consequence the patient visited our outpatient department for DUs management. A global approach including both systemic (incremental use of intravenous prostanoids) and local treatment (advance wound care) was initiated as previously described [15]. A slight reduction in the size of the DUs on II and III fingers was observed over this time, but they continued to cause pain and produce discharge, with bone exposition.

In May 2019, treatment with AFG was performed as additional therapy for the treatment of the stump DUs. Adipose tissue was obtained from the peri-umbilical region after infiltration with a modified Klein solution (50 ml of saline solution, 0.5 ml of 1:1.000 adrenalin and 10 ml of 2% Mepivacain). In detail, fat harvesting was performed using an aspiration cannula (Micro Aspiration Cannulas Black & Black Surgical FAC, 12 gauge × 15 cm Luer-lock), introduced through a 3 mm incision. The required amount of lipoaspirate was estimated using a standard formula: 2 cc lipoaspirate per square centimeter wound size and approximately 15 ml of fat and fluids was aspirated in 10 ml Luer-lock syringes. No centrifugation of the lipoaspirate was performed. The lipoaspirate was then transferred into 1-cc Luer-lock syringes using a closed system (Luer Lock Adapter), followed by infiltration of the lipoaspirate using a 2 × 80-mm Supra Luer Lock cannula into the wound edges and subdermal plane all around the finger stump (crown-shape infiltration). From 0.5 to 1 ml of fat was grafted at least in each finger stump. The finger stump was then covered with a sterile wound dressing. The patient received perioperative and post-operative antibiotic therapy. No side-effects related to the fat grafting injection were recorded and no infections were observed. The only reaction reported was mild pain at the lipoaspiration site, which was rapidly resolved using escin topical cream, and global pain in the ulcer site too (VAS: 6/10) in the days following AFG, requiring transient increase in analgesic therapy (paracetamol, fentanyl). Finger stiffness was also reported after the fat microinjection. The patient displayed neither clinical inflammation nor pain during any of the subsequent weekly visits, merely a mild oedema at the site of the lipoaspirate injection.

**Figure 1.**
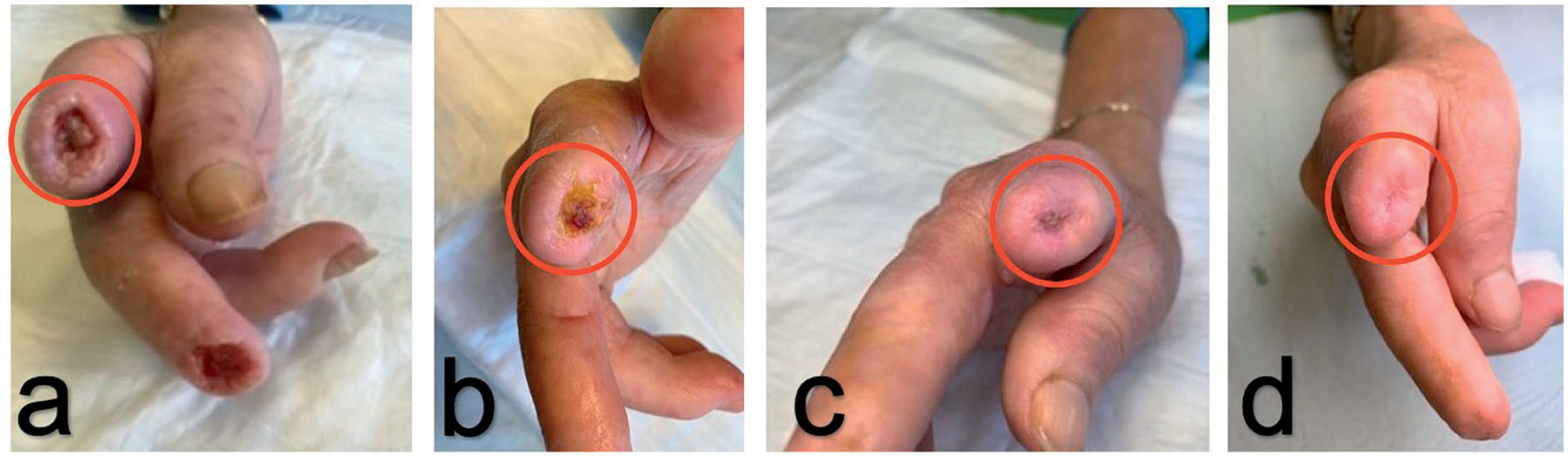
(a–d): Healing process of digital ulcer over stump site (II finger of the right hand) after injection of autologous adipose cells. The circles indicate the site of fat graft as crown-shape infiltration.

**Figure 2.**
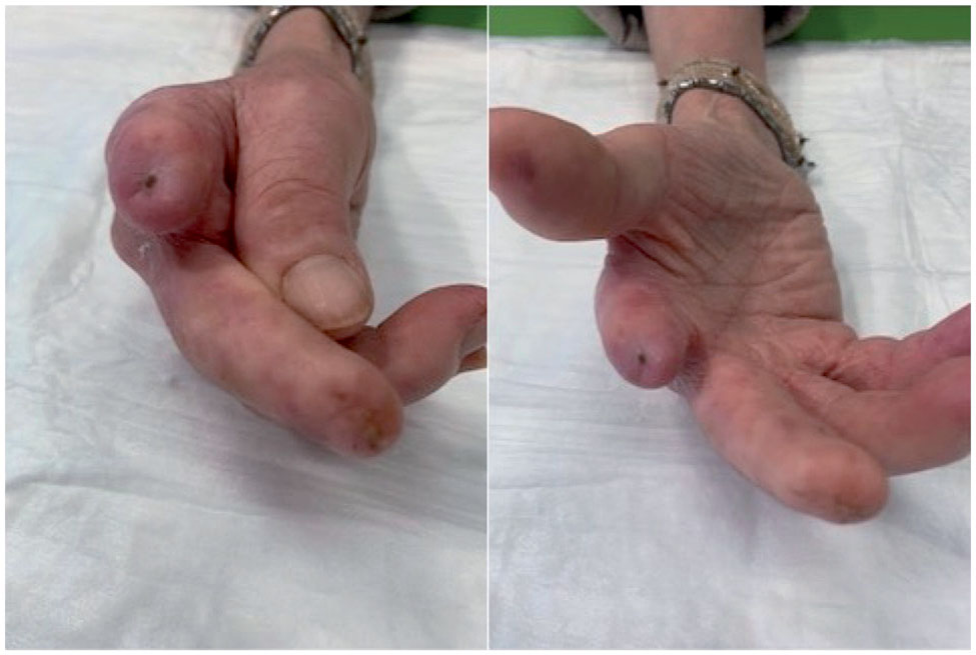
Healing process at 6 months: detail of II and III fingers of the right hand 6 months after injection of autologous adipose cells.

After the first month of AFG treatment, the DUs gradually improved showing re-epithelialization from the margins and granulation of the wound bed; by the end of the 3-month treatment period they had all completely healed. Daily use of analgesics was gradually reduced and discontinued within 2 months, as expression of AFG efficacy in pain management too. These remarkable results remained stable during the 3 and 6 month clinical observations. Three months after AFG, no other amputations were needed (Figure 1(a–d)). The patient is still under regular clinical observation in order to determine the long term effects of AFG (Figure 2).

## Discussion

The results of our report suggested that AFG treatment represents an important approach to the scleroderma stump skin ulcers responsible for severe chronic pain and marked reduction of patients’ quality of life. In addition, the treatment with fat grafting was significantly more effective than systemic vasoactive therapy and traditional topical approach and it was well tolerated. The treatment of scleroderma DUs is challenging, particularly for lesions complicated by local infections and gangrene that compromise the outcome of skin lesions in a significant percentage of patients, often requiring surgical amputation [16]. Stump skin ulcers are difficult to treat and many of treatment are anecdotally supported. As far as we know, this is the first report showing the healing of the stump-DUs in a scleroderma patient using AFG.

Autologous fat grafting is frequently used for soft tissue defect reconstruction and it becomes a relatively common procedure due its availability, low rate of adverse events and relative ease of harvesting. Several modifications have been made to the procedures of fat harvesting, processing, and injecting [17]. Fat tissue is usually obtained from the flanks, peri-umbilical region, or internal side of the thigh or knee. Low negative-pressure aspiration with large-bore cannulas minimize adipocyte damage during fat harvesting. According to other authors in our case was used the ‘wet’ method of fat harvesting which involves fluid injection at the donor site and facilitates lipoaspiration, while minimizing pain and ecchymosis. For fat processing, centrifugation at a low speed is preferable to high-speed centrifugation, gravity separation or filtration [17,18]. No centrifugation of the lipoaspirate was performed in our cases as suggested by other authors to prevent fat damage [19]. After decantation, fat injection was performed using small-gauge cannulas in a fanning out pattern over multiple sessions, rather than a single session. Fat is gently distributed above the dorsal deep fascia to avoid perforation of the vessels. Treatment of stump skin ulcers in scleroderma patients is poorly investigated even though it is a severe and invalidating symptom, mainly in patients with digital gangrene.

The usefulness of AFG in the treatment of stump skin lesions was reported firstly by Bourne et al. [20], and confirmed by Malik et al. [21], in patients with traumatic lower extremity amputations.

The reason why local fat grafting may induce such positive changes in SSc stump skin ulcers remains almost completely speculative at the moment.

Some studies have shown that stem cells in fat tissue are multipotent cells potentially able to differentiate themselves into other cell types, like cells osteogenic, myogenic, endothelial under the conditioning of different stimulant, as demonstrated in a lot of in vitro and in vivo studies. There is also an evidence that progenitor cells may play a role in fat grafting. It is well established that multiple cell types, including mature adipose cells, endothelial progenitor cells, pericytes, and adipose-derived stromal cells are present in harvested adipose tissue [22–24]. Several studies have demonstrated the regenerative powers of AFG, probably related to the production of growth factors such as vascular endothelial growth factor (VEGF), keratinocyte growth factor (KGF) and other factors which lead to keratinocyte migration and acceleration of healing. AFG may also directly stimulate the production from other resident cell types of a number of cytokines with immune-modulating action probably capable of downregulating some pathological mechanisms in injured tissues [25–29]. Since SSc is a systemic endothelial disorder characterized by a reduction of capillary flow and the consequent fibrotic evolution, it could be postulated that AFG may be involved in releasing a number of growth factors and cytokines which stimulate the angiogenesis and the formation of new capillary loops, able to the heal stump DUs as observed in our study.

The present study confirmed previous data suggesting that local transfer of autologous may be a successful option to induce healing in ischemic SSc related fingertip DUs that are resistant to more traditional therapeutic approaches [30,31]. Clinical improvements observed in our patient on DUs after AFG are definitely in line with more recent reports on the treatment of fibrosis of the skin and wound healing [32–37].

In conclusion, our report suggests that the use of AFG is effective and safe for the treatment of SSc manifestations as stump skin ulcers and related pain. Nevertheless, further clinical and experimental studies are needed, in order to better understand the exact mechanisms of action of stem cells and to standardize this approach.
